# Saikosaponin D reverses epinephrine- and norepinephrine-induced gemcitabine resistance in intrahepatic cholangiocarcinoma by downregulating ADRB2/glycolysis signaling: Saikosaponin D reverses gemcitabine resistance

**DOI:** 10.3724/abbs.2023040

**Published:** 2023-07-25

**Authors:** Hui He, Jiaqi Guo, Yunxiang Hu, Han Zhang, Xinyang Li, Jian Zhang, Shi Jin

**Affiliations:** 1 Department of Laparoscopic Surgery the First Affiliated Hospital of Dalian Medical University Dalian 116000 China; 2 Department of Interventional Therapy the First Affiliated Hospital of Dalian Medical University Dalian 116000 China

**Keywords:** saikosaponin D, gemcitabine resistance, intrahepatic cholangiocarcinoma, ADRB2, glycolysis

## Abstract

Intrahepatic cholangiocarcinoma (iCCA) is a highly fatal malignancy with rapidly increasing incidence and mortality worldwide. Currently, gemcitabine-based systemic chemotherapy is the main clinical therapeutic regimen; however, its efficacy is poor, and its mechanism has not been elucidated. In this study, we use a Seahorse Extracellular Flux analyser to measure glycolysis capacity (extracellular acidification rate, ECAR) and oxygen consumption rate (OCR). The glucose uptake or lactic acid content is detected, and the effects of saikosaponin D, an active compound derived from *Bupleuri* *Radix* (a traditional Chinese medicine for soothing the liver and relieving depression), on gemcitabine cytotoxicity in norepinephrine-stimulated iCCA cells are analysed. We find that adrenergic signaling plays a fundamental role in chronic stress-induced therapeutic resistance in iCCA. Norepinephrine (NE) and epinephrine (E) enhance the proliferation of iCCA cells and interfere with the response to gemcitabine through activation of the β2-adrenergic receptor (ADRB2). Furthermore, we find that NE upregulates the expressions of several drug efflux-related genes (such as *ABCG2* and *MDR1*) and promotes glycolysis in iCCA cells. In addition, saikosaponin D reverses the poor response of iCCA cells to gemcitabine by downregulating ADRB2 level. Furthermore, saikosaponin D inhibits drug efflux and glycolysis in iCCA cells by regulating the expressions of MDR1, ABCG2, HK2, and GLUT1. Collectively, saikosaponin D enhances the antitumor effect of gemcitabine by controlling glucose metabolism and drug efflux by inhibiting the ADRB2 signaling. Therefore, the combination of saikosaponin D and gemcitabine may be a potential therapeutic strategy for the treatment of iCCA.

## Introduction

Intrahepatic cholangiocarcinoma (iCCA) is one of the most aggressive forms of cancer and is the second most common primary hepatic malignancy. The incidence and mortality of iCCA have been rapidly increasing worldwide [1]. Surgical resection remains the most efficient treatment for patients with iCCA; however, the tumors are often quite advanced at the time of diagnosis and surgical resection is not usually possible [2]. Moreover, gemcitabine-based systemic chemotherapy [3], which is the main clinical therapeutic regimen, has poor efficacy [4]. This has been mainly attributed to a poor understanding of the molecular mechanisms of this malignancy. Therefore, elucidating the underlying mechanisms of iCCA progression is crucial to improving patient prognosis and survival.

Social and psychological stress is inevitable in our daily lives, which changes neurochemistry and endocrine and immune functions by activating the sympathetic nervous system and releasing neurotransmitters such as catecholamines [5]. Several experimental animal studies have conclusively demonstrated that psychosocial factors, especially chronic stress, can modulate the growth, progression, and therapeutic resistance of certain tumors by inducing the release of neurotransmitters [ 6, 7]. Epinephrine (E) and norepinephrine (NE) are important neurotransmitters in the adrenergic system, which regulate several processes, such as cardiovascular function and smooth muscle tone; they also contribute to the modulation of several tumors [ 8, 9]. iCCA cell lines highly express the α-2A, α2B, and α-2C subtypes of adrenergic receptors, indicating that adrenergic signaling is crucial in iCCA [10]. In addition, the expression of β-2 adrenergic receptor (ADRB2) is significantly higher in iCCA cells than in less invasive tumor cells, thereby facilitating nervous and lymphatic metastasis [11]. Psychological burden is high in patients with hepatobiliary cancers; this is reflected by high levels of depression and anxiety as well as reduced quality of life, which partially affects the therapeutic efficacy of drugs [12]. However, the role of the adrenergic system in the therapeutic response of iCCA has received little attention.

The classic mechanism of chemoresistance in cancer cells involves proteins of the ATP-binding cassette (ABC) transporter family [13]. Currently, 49 ABC transporter subtypes have been identified; of these, ABC subfamily G, isoform 2 protein (ABCG2), and multidrug resistance protein 1 (MRP1) have been implicated in the resistance of cholangiocarcinoma cells to chemotherapeutic agents (such as 5-fluorouracil, taxane derivatives, and doxorubicin) [ 14 , 15]. ABC transporters consist of four domains: two cytoplasmic nucleotide-binding domains that bind and hydrolyse ATP and two transmembrane domains that recognize and transport substrates. When a substrate approaches the cell, the transmembrane domain of the ABC transporter binds to the substrate molecule and undergoes an ATP-dependent conformation change to efflux the substrate out of the cell, reducing its intracellular level [16]. The effect of stress hormones on the role of ABC transporters in chemoresistance has been reported in previous studies [ 17 , 18].

The role of ATP is critical for the activities of these transporters; therefore, it is particularly important to find the source of ATP in cancer cells. Malignant cells increase the expressions of glycolytic enzymes, such as glucose transporter 1 (GLUT1), pyruvate kinase M2 (PKM2), and hexokinase 2 (HK2), to markedly enhance aerobic glycolysis (the Warburg effect), which leads to the production of a large amount of ATP and biomass, such as nucleic acids and lipids, and is essential for cell survival and division [ 19, 20]. Therefore, increased aerobic glycolysis is considered a hallmark of cancer and is the fundamental principle of [18F]-fluorodeoxyglucose-positron emission tomographic imaging used in clinical settings to detect malignant tissues [21]. The inhibition of glycolysis preferentially targets malignant cells in leukemia, myeloma, hepatocellular carcinoma, and breast carcinoma to suppress ATP synthesis [ 22– 24]. Reduced ATP levels can inactivate ABC transporters, and anticancer agents can be retained intracellularly to restore their cytotoxic effects on cancer cells [25]. Several diseases, such as septic shock, memory impairment, and cancer, are characterized by the overexpression of NE and E, which can induce aerobic glycolysis and contribute to the progression of the disease [ 26 – 28].

Herbal and natural products are important sources of anticancer drugs. Some natural compounds or their derivatives, such as Taxol, vinblastine, camptothecin, and etoposide, have been used for the treatment of cancer [29]. *Radix Bupleuri* (Chai Hu in the Chinese language) is a famous traditional Chinese medicine that has been used to treat influenza, hyperlipidemia, menstrual disorders, liver disease, and depressive or anxiety disorders [30]. *Radix Bupleuri* can regulate the level of NE in patients with anxiety or depression [31]. Several active ingredients extracted from its roots, consisting of triterpenoid glycosides (saikosaponins), polyacetylenes, flavonoids, lignans, and essential oils, possess antitumor activities against multiple cancers. Among them, saikosaponin D (SSD) inhibits the proliferation of various cancer cells, which might be associated with the induction of apoptosis, suppression of TNF-α-induced NF-κB activation, autophagic cell death, or inactivation of the Wnt/β-catenin signaling pathway [ 32– 35]. In addition, SSD has also been shown to reverse chemoresistance by regulating the activity of multiple ABC transporters [36]. However, the role of SSD in enhancing the sensitivity of cancer cells to therapeutic drugs by regulating the adrenergic receptors activated by neurotransmitters has not been reported. In addition, the anticancer activity of SSD in iCCA has not been explored.

In the present study, we investigated the role of neurotransmitters (NE or E) and their receptors in iCCA cells with poor response to gemcitabine. Furthermore, the mechanisms of therapeutic resistance triggered by ADRB2 activation were investigated. Finally, we explored the mechanism of SSD-mediated reversal of NE- and E-induced gemcitabine resistance in iCCA cells.

## Materials and Methods

### Cell culture and reagents

The human iCCA cell lines RBE and HuCCT1 were purchased from Shanghai Bioleaf Biotech Co. Ltd. (Shanghai, China). The two iCCA cell lines were cultured in RPMI 1640 medium (Gibco, Carlsbad, USA) supplemented with 10% fetal bovine serum (FBS; Gibco) and 100 U/mL penicillin (Gibco) at 37°C with 5% CO _2_. The cell lines were identified by short tandem repeat typing. Primary antibodies against ADRB2 (#8513), ABCG2 (#42078), MRP1 (#72202), HK2 (#2867), and GLUT1 (#73015) were purchased from Cell Signaling Technology (Danvers, USA). Epinephrine and norepinephrine were purchased from Sigma-Aldrich (St Louis, USA). SSD and ICI118551 were purchased from MedChemExpress (Monmouth Junction, USA).

### Cell proliferation and colony formation assay

Cell proliferation was measured using CCK8 kit (Dojindo, Tokyo, Japan) according to the manufacturer’s instructions. Cells treated with various ADRB agonists, gemcitabine, and SSD were seeded at a density of 1×10 ^4^ cells/well in a 96-well flat-bottom plate and cultured for CCK8 assay according to the manufacturer’s protocol. Cell viability was detected at 0, 24, 48, 72, and 96 h. For the colony formation assay, cells were plated in 6-well plates at 500 cells/well and cultured in RPMI-1640 medium containing 10% FBS. After 2 weeks, the plates were washed with phosphate-buffered saline (PBS) and stained with crystal violet for 15 min, and the number of colonies were counted. All experiments were performed in triplicate.

### Flow cytometry analysis

The cells were digested with 0.05% trypsin and washed three times with PBS. Next, the cells (1×10 ^6^ cells) were resuspended in binding buffer and then incubated with 5 μL Annexin V-FITC and 10 μL propidium iodide (Sigma-Aldrich) for 15 min at room temperature (25°C). The fluorescence signal at 488/530 nm was recorded using a flow cytometer.

### Western blot analysis

Western blot analysis was performed as previously described [8]. In brief, whole-cell extracts were sonicated in lysis buffer and homogenized. Samples containing 30–50 μg total protein were resolved on 8%–12% SDS-polyacrylamide gels and electrophoretically transferred onto polyvinylidene difluoride (PVGF) membranes. The membranes were blocked with 5% skimmed milk, incubated with primary antibodies, and finally incubated with an HRP-conjugated secondary antibody. Protein bands were detected using a Chemiluminescence kit (Roche, Basel, Switzerland).

### Reverse transcription-polymerase chain reaction (RT-PCR)

Total RNA was isolated using Trizol (Invitrogen, Carlsbad, USA), and cDNA was synthesized using PrimeScript RT reagent (TaKaRa, Dalian, China) according to the manufacturer’s protocol. cDNA was amplified using Power SYBR Green PCR master mix (Applied Biosystems, Foster City, USA) according to the manufacturer’s protocol on an Applied Biosystems 7500 sequence detection system. The gene expression levels were determined using the 2 ^–ΔΔCT^ method, and the results were expressed as mRNA expression level normalized to that of *β-actin*. The qRT-PCR primers used were as follows: *ADRB2*, 5′-TTGCTGGCACCCAATAGAAGC-3′ (forward) and 5′-CAGACGCTCGAACTTGGCA-3′ (reverse); and *β-actin*, 5′-TTAGTTGCGTTACACCTTTC-3′ (forward) and 5′-ACCTTCACCGTTCCAGTTT-3′ (reverse). Each PCR analysis was performed in triplicate and independently repeated three times.

### RNA interference and lentivirus transfection

Lentiviral vectors containing shRNA against ADRB2 (shADRB2-1: 5′-TGAGACCTGCTGTGACTTCTT-3′, shADRB2-2: 5′-GGCAACTTCTGGTGCGAGTTT-3′) and negative control shRNA (shNTC: 5′-GGAATCTCATTCGATGCATAC-3′) were prepared by GeneChem Corporation (Shanghai, China). The concentrated virus was added to the two iCCA cell lines (RBE and HuCCT1) grown in 6-well plates and incubated for 12 h. Spectinomycin (Clontech, Mountain View, USA) was used to select lentivirus-transfected iCCA cells.

### Metabolic analysis

Glycolysis capacity (extracellular acidification rate, ECAR) and oxygen consumption rate (OCR) were measured using a Seahorse Extracellular Flux analyser (XF-24; Seahorse Biosciences, Shanghai, China) according to the manufacturer’s protocol. Briefly, iCCA cells were cultured, and approximately 50,000 cells/well were plated into XF24 V7 PS cell culture microplates (09516; Seahorse Biosciences) and incubated for 12 h at 37°C in a 5% CO _2_ humidified atmosphere. Cells were washed with the XF assay medium and were then kept in the same medium at 37°C in a non-CO _2_ incubator for 1 h. For the measurement of OCR, 2 μM oligomycin, 5 μM FCCP, 2 μM antimycin A, and rotenone were sequentially loaded into the injection ports in the Xfe 96 sensor cartridge. For the measurement of ECAR, within the incubation time period, 10 mM glucose, 1 μM oligomycin, and 20 mM 2-deoxyglucose were sequentially loaded into the injection ports in the XFe 96 sensor cartridge.

### Measurement of glucose uptake and lactic acid content

The concentration of glucose in the supernatant was measured using a standard kit (K686; BioVision, San Francisc, USA) according to the manufacturer’s instructions. Briefly, iCCA cells were seeded in 6-well plates. After the cells were attached, the cell culture medium was discarded and replaced by fresh medium containing different concentrations of NE and SSD and incubated for 24 h. The supernatant in the 6-well plate was collected. The absorbance was measured using an automatic biochemical analyser (7170A; Hitachi, Tokyo, Japan). The relative glucose consumption rate was normalized by the protein concentration of the samples.

The concentration of lactic acid, an important indicator of carbohydrate metabolism and aerobic metabolism, in the cell supernatant weas measured using a standard kit (K627; BioVision) according to the manufacturer’s instructions. Briefly, iCCA cells were seeded in 6-well plates. After the cells were attached, the cell culture medium was discarded and replaced by fresh medium containing different concentrations of NE and SSD and incubated for 24 h. The supernatant in the six-well plate was collected. The absorbance was measured using an automatic biochemical analyser.

### Statistical analysis

Data were analysed using SPSS 21.0 and presented as the mean±SD unless otherwise indicated. If normally distributed, continuous variables between two groups were compared with Student’s *t* test, whereas comparisons among three or more groups were performed with one-way analysis of variance (ANOVA) followed by Tukey’s test. Otherwise, nonparametric tests, such as Tukey’s test (if appropriate), were used to compare differences. Differences were considered statistically significant at *P*<0.05.

## Results

### Inhibition of ADRB2 reduced proliferation and NE-induced gemcitabine resistance in iCCA cells

**Figure 1:**
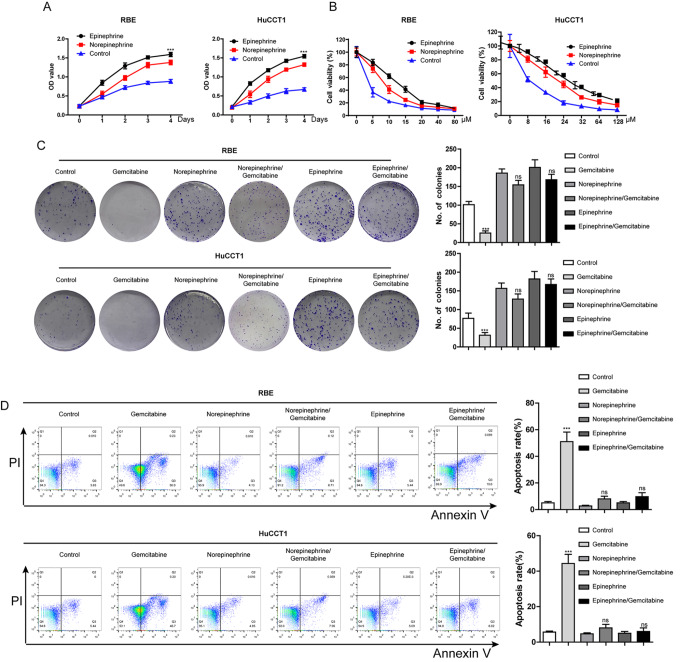
Figure 1 Effects of E and NE on the proliferation and gemcitabine resistance of iCCA cells (A) Effects of NE and E on RBE and HuCCT1 cell proliferative ability were determined by CCK8 assay and presented as a growth curve (***P<0.001 vs control). (B) Effects of NE and E on the inhibition of iCCA cells induced by gemcitabine. (C) Colony formation assay was used to detect the effects of NE and E with/without gemcitabine on the colony-forming ability of RBE and HuCCT1 cells. Representative images are shown on the left (ns, no significant vs E or NE alone). (D) Effects of NE and E on gemcitabine-induced apoptosis in iCCA cells (ns, no significant vs E or NE alone). Data are presented as the mean±SD ( n=3).

First, we treated RBE and HuCCT1 cells with epinephrine (10 μM) and norepinephrine (10 μM) for 1, 2, 3 or 4 days. The viability of the two cell lines was markedly increased after treatment ( Figure 1A). Furthermore, the colony formation ability of the iCCA cell lines was also enhanced after treatment with epinephrine and norepinephrine ( Figure 1C). Finally, the CCK8 assay showed that epinephrine and norepinephrine notably inhibited the killing effect of gemcitabine on RBE and HuCCT1 cells and increased the IC _50_ of gemcitabine ( Figure 1B). Similar results were obtained in the subsequent colony formation and apoptosis assays ( Figure 1C,D). Therefore, epinephrine and norepinephrine can significantly reverse the inhibitory effect of gemcitabine on iCCA cell growth and apoptosis induction.

**Figure 2:**
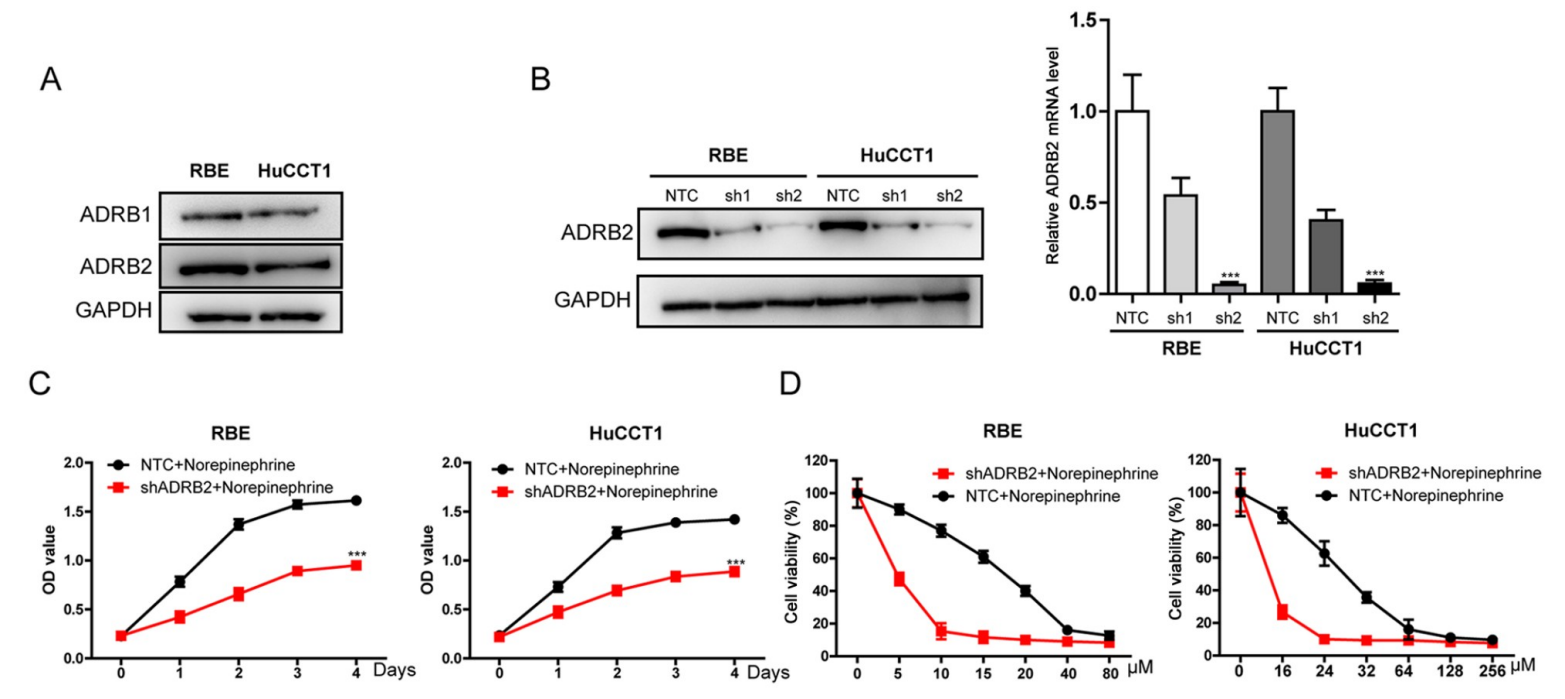
Figure 2 Effect of ADRB2 on the proliferation and gemcitabine resistance of iCCA cells (A) Western blot analysis was used to analyze the expression level of ADRB proteins in RBE cells and HuCCT1 cell lines. (B) The efficiency of ADRB2 knockout was determined by real-time quantitative PCR and western blot analysis (***P<0.001 vs NTC). (C) Effects of ADRB2 knockout on NE-stimulated iCCA cell proliferation by CCK8 assay and are presented as a growth curve (*** P<0.001 vs norepinephrine+NTC). (D) Effects of ADRB2 knockout on NE-induced gemcitabine resistance in iCCA cells. Data are presented as the mean±SD (n=3).

To elucidate the mechanism by which NE and E affect the growth and treatment resistance of iCCA cells, the expression levels of their receptors in cancer cells were determined. Our results indicated that the human iCCA cell lines RBE and HuCCT1 express both ADRB1 and ADRB2 adrenergic receptors ( Figure 2A). The expression of ADRB2 receptor was approximately 5-fold higher than that of ADRB1 receptor in the two cell lines. These results suggest that ADRB2 may be the most important adrenergic receptor in the iCCA cell lines.

Furthermore, we introduced the shADRB2 or control shNTC into lentiviruses and infected cells to establish stable *ADRB2*-knockdown (RBE ^shADRB2^ and HuCCT1 ^shADRB2^) cell lines to verify the function of ADRB2 in iCCA. The effect of *ADRB2* knockdown in the cell lines was determined by RT-PCR and western blot analysis. The shADRB2 reduced the mRNA level of ADRB2 by 90% ( Figure 2B). After confirming the knockdown effect, the two cell lines were treated with NE. The growth-promoting activities of NE on cancer cells were significantly inhibited in the RBE ^shADRB2^ and HuCCT1 ^shADRB2^ cells ( Figure 2C). In addition, inhibition of ADRB2 expression significantly attenuated NE-induced gemcitabine resistance compared with that in the NTC group ( Figure 2 D).

### ADRB2 enhanced aerobic glycolysis and related metabolic processes in iCCA cells

**Figure 3:**
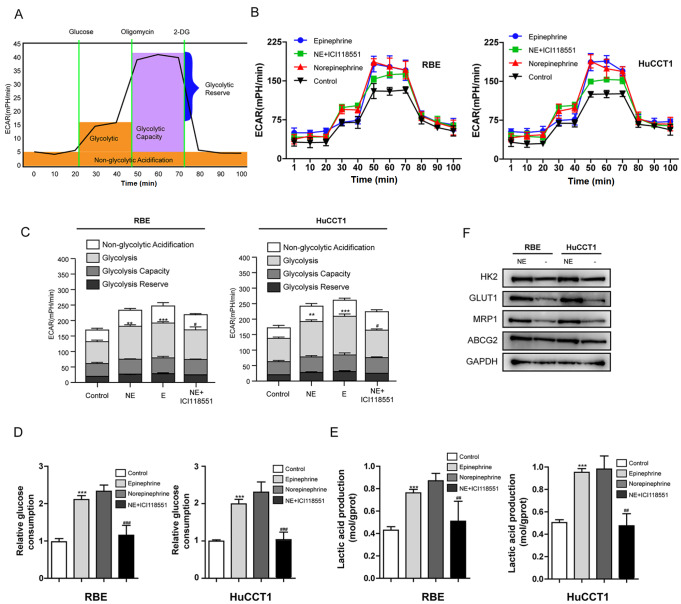
Figure 3 Effects of ADRB2 on aerobic glycolysis and ABC transporter levels in iCCA cells (A,B) Measurement of the extracellular acidification rate (ECAR) in iCCA cells influenced by NE, E and ICI118551 with the XFe24 Extracellular Flux Analyser. (C) Glycolytic variations (glycolysis, glycolytic capacity and glycolytic reserve) in each group were summarized from raw data (**P<0.01, ***P<0.001 vs control; #P<0.05 vs NE). (D) Glucose consumption in each group was detected using a glucose assay kit (***P<0.001 vs control; ###P<0.001 vs NE). (E) Production of lactic acid in each group was assayed using a Lactic acid production detection kit (***P<0.001 vs control; ##P<0.01 vs NE). (F) Western blot analysis was used to analyse the protein expression levels of HK2, GLUT1, ABCG2 and MRP1in RBE cells and HuCCT1 cell lines from each group. Data are presented as the mean±SD (n=3).

We conducted bioenergetic profiling of iCCA cells with the Seahorse XF Bioanalyzer Platform to determine whether overproliferation and gemcitabine resistance in iCCA cells after ADRB2 activation is associated with metabolic differences (potential Warburg effect). Tumor cells rely on high glycolysis rate to meet their rapid, uncontrolled proliferation requirements and other energy needs. We observed the glycolytic activity of iCCA cells by monitoring the real-time changes in ECAR ( Figure 3A). Compared with the untreated cells, the basal ECAR was enhanced in iCCA cells treated with NE and E ( Figure 3B,C). Oxidative phosphorylation was inhibited, and acid production was increased after the addition of oligomycin. The increased ECAR suggested that the cells had inherent glycolytic ability, and NE and E could promote this maximum potential ( Figure 3B,C). 2-Deoxyglucose is a glycolysis inhibitor, and the increase in ECAR indicated that a mechanism other than glycolysis is involved. After NE and E treatment, the high glycolysis reserve capacity of the cells indicated that these cells operated at their general glycolysis rate and had the ability to increase their glycolysis flux in response to additional metabolic stress ( Figure 3B,C). In addition, the conversion of glucose to lactic acid, glucose consumption, and lactate production were measured to reflect the glycolysis level. The results showed that NE and E treatment markedly increased glucose consumption and lactate production in RBE and HuCCT1 cells ( Figure 3D,E). Furthermore, NE upregulated the levels of core glycolytic enzymes (HK2 and GLUT1) in iCCA cells ( Figure 3F). However, ICI118551 (a selective ADRB2 antagonist) reversed NE-induced glycolysis in both RBE and HuCCT1 cells ( Figure 3B–E). Taken together, these results indicated the enhanced effect of ADRB2 on glycolysis and related metabolic processes in iCCA cells.

### Norepinephrine upregulated the expressions of drug efflux-related genes in iCCA cells

Overproliferation and gemcitabine resistance in iCCA cells could be associated with the expressions of drug efflux-related genes. Therefore, western blot analysis was used to measure the changes in the expressions of ABCG2 and MRP1 before and after NE treatment. Stimulation of the iCCA cell lines with 10 μM NE for 0.5 h transiently increased the expression of ABCG2 (1.5 folds) in the iCCA cells ( Figure 3F). Similarly, MRP1 expression was increased (3.6 folds) after NE treatment in both cell lines at the same time point ( Figure 3F).

### Saikosaponin D enhanced gemcitabine cytotoxicity in iCCA cells stimulated with NE

**Figure 4:**
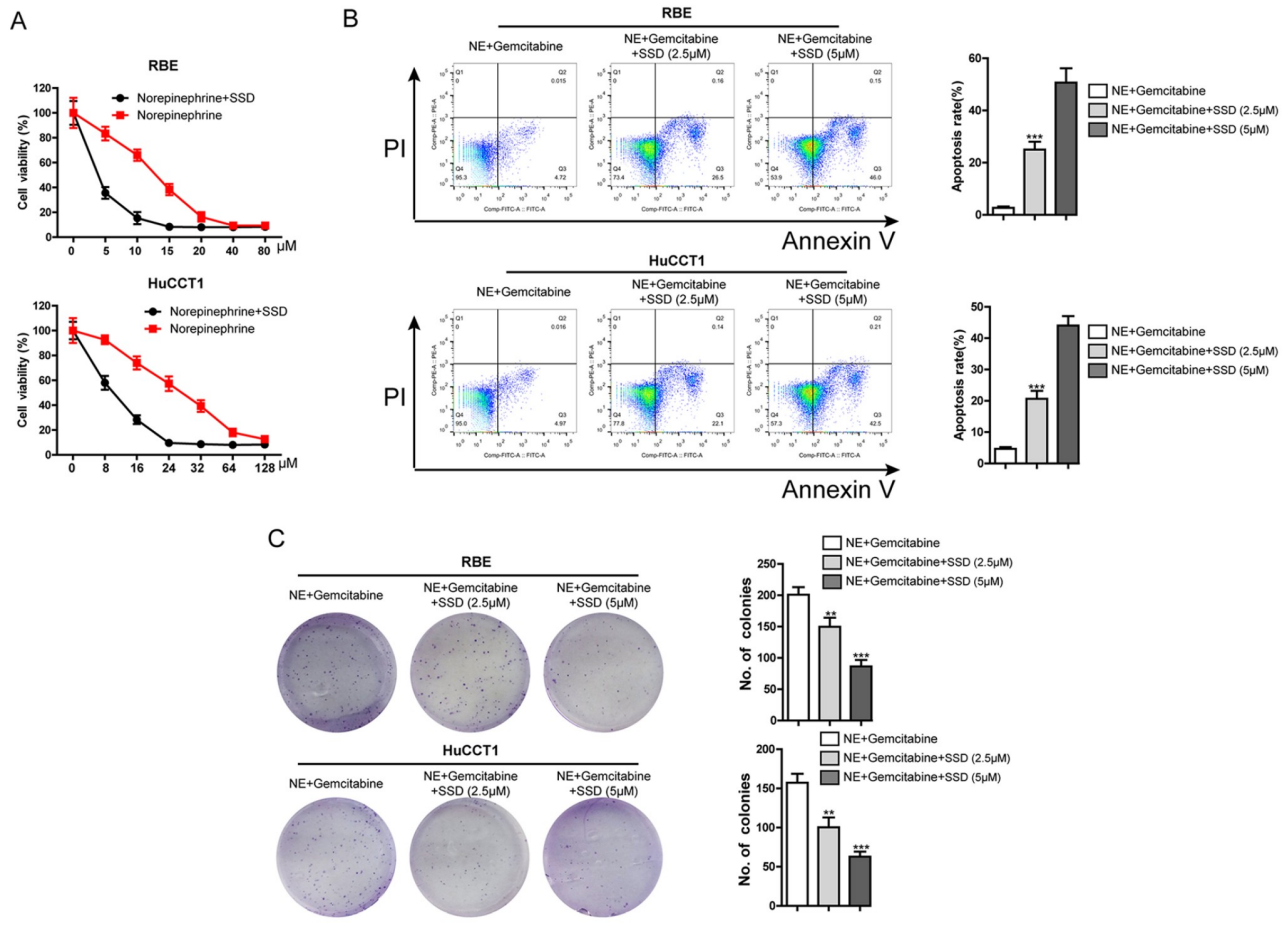
Figure 4 Effects of SSD on the proliferation and gemcitabine resistance of iCCA cells induced by NE (A) Effects of SSD on NE-induced gemcitabine resistance in iCCA cells. (B) Colony formation assay was used to detect the effects of NE and gemcitabine with/without SSD (2.5 and 5 μM) on the colony-forming ability of RBE and HuCCT1 cells. Representative images are shown on the left (**P<0.01 vs NE+gemcitabine). (C) Effects of NE and gemcitabine with/without SSD (2.5 and 5 μM) on apoptosis in RBE and HuCCT1 cells (***P<0.001 vs NE+ gemcitabine). Data are presented as the mean±SD (n=3).

The iCCA cells were randomly divided into three groups and then treated with NE+gemcitabine and NE+gemcitabine+SSD (2.5 μM). Then a CCK-8 cell proliferation kit was used to detect cell viability after 24 h. The administration of SSD significantly inhibited cell viability compared with gemcitabine administration alone ( Figure 4A). Therefore, we inferred that SSD could enhance the chemotherapeutic effect of gemcitabine on iCCA. Furthermore, we randomly divided RBE and HuCCT1 cells into three groups and then treated them with NE+gemcitabine, NE+gemcitabine+SSD (2.5 μM), and NE+gemcitabine+SSD (5 μM). Cell apoptosis was detected by flow cytometry and Annexin V/PI staining after 24 h. The results showed that the combined treatment with SSD and gemcitabine significantly increased the apoptosis rate compared with treatment with gemcitabine alone ( Figure 4B). Moreover, colony formation assay revealed that SSD (2.5 and 5 μM) enhanced the inhibitory effect of gemcitabine in iCCA cells stimulated with NE ( Figure 4C). This phenomenon was dependent on SSD concentration ( Figure 4B,C).

### Saikosaponin D inhibited ADRB2/glycolysis signaling in iCCA cells stimulated with NE

**Figure 5:**
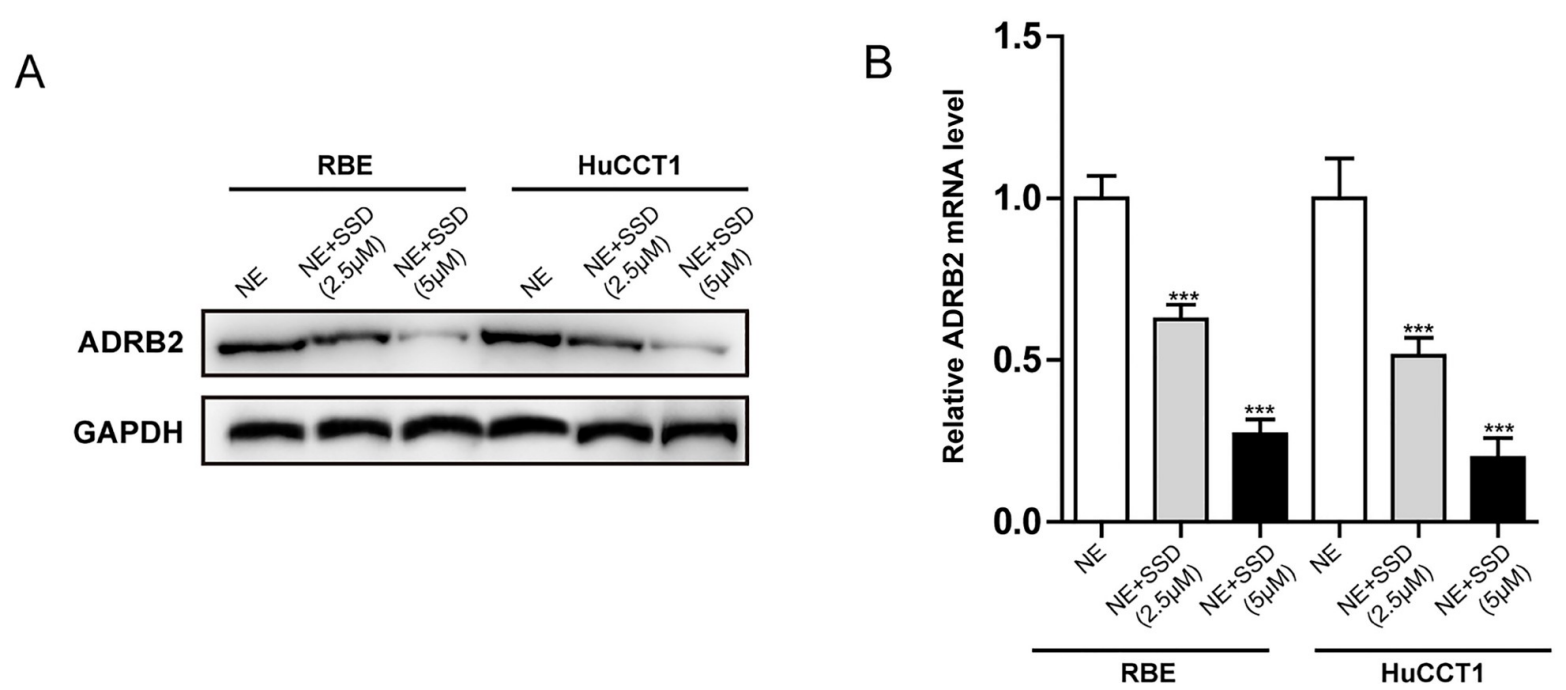
Figure 5 Effect of SSD on ADRB2 expression in iCCA cells stimulated with NE The effects of SSD on the levels of ADRB2 were determined by western blot analysis (A) and real-time quantitative PCR (B). Data are presented as the mean±SD (n=3). ***P<0.001 vs NE.

**Figure 6:**
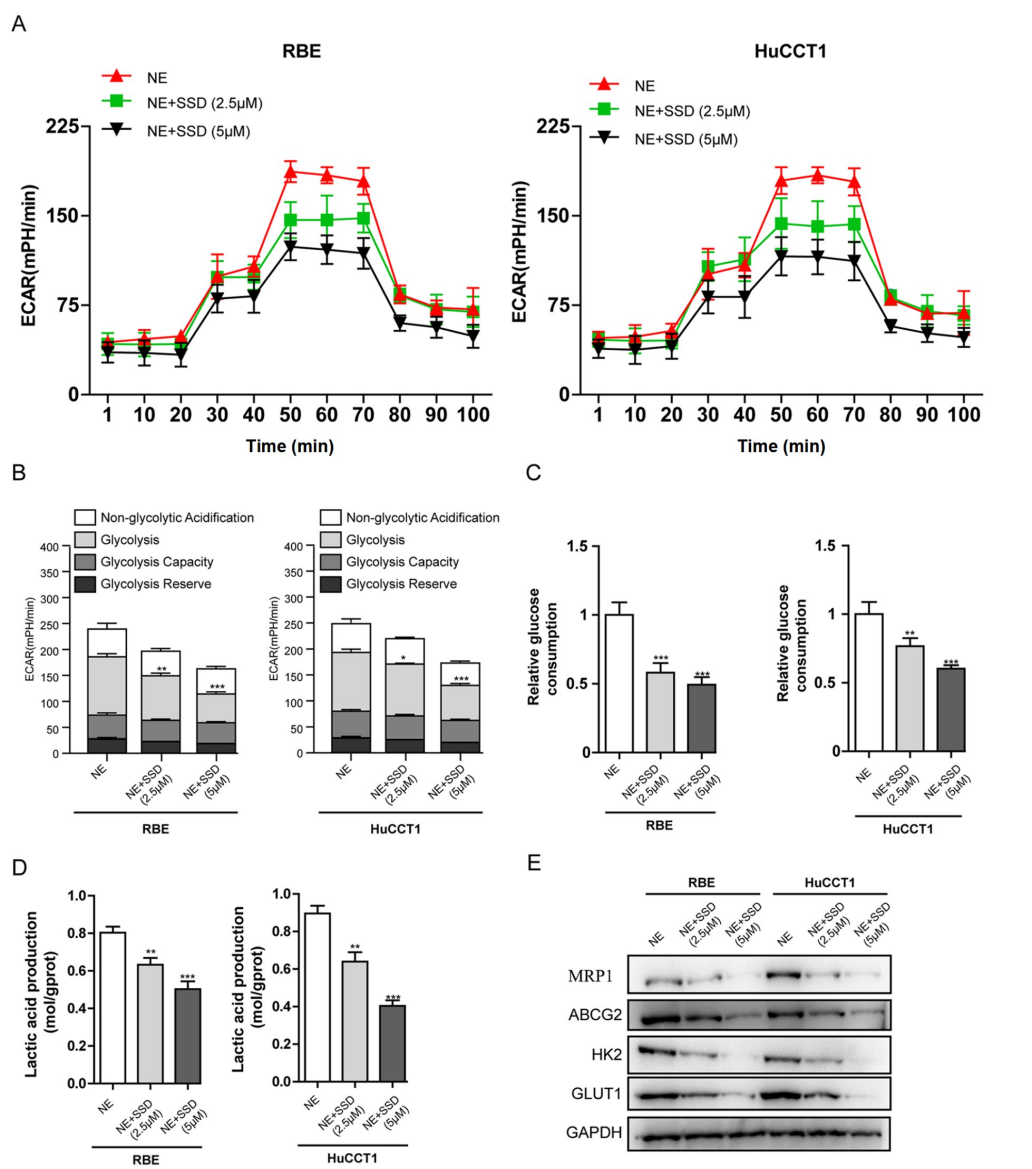
Figure 6 Effect of SSD on ADRB2/glycolysis signaling in iCCA cells stimulated with NE (A) Measurement of the extracellular acidification rate (ECAR) in iCCA cells influenced by NE with/without SSD (2.5 and 5 μM) with the XFe24 Extracellular Flux Analyzer. (B) Glycolytic variations (glycolysis, glycolytic capacity and glycolytic reserve) in each group were summarized from raw data (*P<0.05, **P<0.01, ***P<0.001 vs NE). (C) Glucose consumption in each group was detected using a glucose assay kit (**P<0.01, ***P<0.001 vs NE). (D) Production of lactic acid in each group was assayed using a Lactic acid production detection kit (**P<0.01, ***P<0.001 vs NE). (E) Western blot analysis was used to analyse the protein expression levels of HK2, GLUT1, ABCG2 and MRP1 in RBE cells and HuCCT1 cell lines from each group. Data are presented as the mean±SD ( n=3).

We first explored the regulatory effect of SSD on ADRB2 expression to elucidate the mechanism by which SSD enhances the therapeutic effect of gemcitabine in iCCA. SSD inhibited the expression of ADRB2 at the protein and mRNA levels in RBE and HuCCT1 cells stimulated with NE in a dose-dependent manner ( Figure 5A,B). In addition, to determine whether metabolic differences are associated with the inhibitory effect of SSD on ADRB2 expression, we measured the glycolytic activity of iCCA cells treated with NE and different concentrations of SSD (0, 2.5, and 5 μM) through monitoring the real-time changes in ECAR. Compared with the NE-treated cells, the basal ECAR was decreased in iCCA cells treated with SSD along with NE ( Figure 6A,B). Oxidative phosphorylation was inhibited, and acid production was enhanced after the addition of oligomycin. The increased production indicated that the cell also had glycolytic ability, and ECAR profiles showed that SSD could inhibit this maximum potential ( Figure 6A,B). After treatment with SSD, the low glycolysis reserve capacity of the cells indicated that these cells operated at their maximum glycolysis rate and had the ability to increase their glycolysis flux in response to additional metabolic stress ( Figure 6A,B). In addition, we also detected the conversion of glucose to lactic acid, glucose consumption, and lactate production to measure the glycolysis level in RBE and HuCCT1 cells treated with or without SSD. Our results showed that SSD treatment notably reduced glucose consumption and lactate production in iCCA cells in a dose-dependent manner ( Figure 6C,D). Furthermore, SSD also downregulated the levels of core glycolytic enzymes (HK2 and GLUT1) and drug-efflux transporters (ABCG2 and MRP1) in iCCA cells stimulated with NE ( Figure 6E).

## Discussion

We concluded that sympathetic innervation is positively correlated with the therapeutic resistance of gemcitabine in iCCA and that sympathetic neurotransmitters (NE and E) promote the malignancy of iCCA cells by activating ADRB2. Our results revealed the supportive role of sympathetic innervation in the pathogenesis of iCCA and suggested ADRB2 as a potential therapeutic target for the treatment of iCCA. Sympathetic nerves infiltrate the tumor microenvironment and actively stimulate cancer cell growth, dissemination, and treatment resistance. This mechanism involves the release of neurotransmitters, such as catecholamines and acetylcholine, directly into the vicinity of cancer and stromal cells to activate the corresponding membrane receptors. Denervation can inhibit the occurrence of digestive system neoplasms, which is linked to the inhibition of the Wnt signaling pathway and the expansion of cancer stem cells [37]. In addition, activation of muscarinic receptors has been suggested to promote cell transformation and cancer progression [38]. Therefore, this study supports the theory of autonomic nervous system regulation of iCCA progression.

We provided conclusive evidence that the stress hormones NE and E undermine the response of iCCA cells to gemcitabine. Chemoresistance is a consequence of different processes that act together to inactivate or block the entry of the drug into malignant cells. ABCG2 and MRP1 have been identified as the major molecules mediating the resistance to several drugs, including cisplatin, 5-fluorouracil and gemcitabine, in different types of cancer. We found that ABCG2 and MRP1 expressions were increased in both cell lines induced by NE. These results suggest that NE may activate the transmembrane proteins ABCG2 and MRP1 in iCCA cells, which pump out the gemcitabine molecule, leading to drug resistance.

Glycolysis could be a target to overcome chemoresistance in cancer patients [39]. Moreover, drug resistance induced by drug efflux transporters is closely related to ATP synthesis, which relies on glycolysis in cancer cells. Li *et al*. [40] developed a simple, versatile, and efficacious self-assembled cyclometalated ruthenium complex, RuZ, which can inhibit mitochondrial respiration and oxygen glycolysis in multidrug resistant cancer cells, markedly decrease intracellular ATP level and result in the inactivity of efflux pumps, thereby overcoming therapeutic resistance. Endothelial cells, lymphocytes, and myeloid-derived suppressor cells are regulated by neurotransmitters (such as NE and E) in the tumor microenvironment, which affects intracellular glycolysis and regulates cell function [ 28, 41]. However, little is known about the relationship between ADRB2 activation and aerobic glycolysis in tumor cells. Here, we showed for the first time that ADRB2 activation by neurotransmitters can reduce the intracellular accumulation of gemcitabine and can induce gemcitabine resistance by promoting aerobic glycolysis and its downstream drug-efflux transporters. ABCG2 and MRP1, as the proteins of the ATP-binding cassette (ABC) transporter family, participate in the process of cancer cell chemotherapy resistance. Since ABCG2 and MRP1 are transfer proteins dependent on ATP, ATP is required to play a role. As a key source of ATP in tumor cells, the level of glucose is critical. After NE/E stimulation, the anaerobic glucolysis capabilities of tumor cells are enhanced to produce a large amount of ATP, which produces resistance to drug resistance through the action of ABCG2 and MRP1. Our data indicate that through the ADRB2/glycolysis signaling pathway, SSD can markedly induce anaerobic glucolysis stimulated by E and NE and then inhibit ATP synthesis, which decreases the levels of ABCG2 and MRP1 and affects the functions of ABCG2 and MRP1. SSD is one of the active compounds present in *Bupleuri Radix*, a representative medicine with an effect on soothing the liver and relieving depression, which is used for the treatment of various liver diseases and depressive or anxiety disorders in traditional Chinese medicine. SSD exhibits a promising antitumor effect in various types of cancers, including lung, liver, breast, and prostate cancers. Studies have shown that SSD can reverse drug resistance of some cancer cells while inhibiting the growth of other cancer cells. In non-small cell lung cancer, SSD and GEFITINIB are combined to reduce the expressions of P-Stat3 and BCL2, indicating that SSD can inhibit the activation of the P-Stat3/BCL2 signaling pathway induced by gefitinib and inhibit the resistance of gefitinib in cancer [42]. In breast cancer, SSD can enhance the sensitivity of MCF-7/ADR cells to ADR by lowering the expressions of MDR1 and P-GP in cancer cells. SSD reduces the expressions of MDR1 and P-GP, which participates in the reversal of MDR [36]. Similarly, in glioblastoma, SSD can partially inhibit the malignant phenotype of LN-229 cells and increase the apoptosis of LN-229 cells and lactate dehydrogenase (LDH) release after treatment with TMZ. At the same time, SSD can improve the chemotherapy effect of TMZ by inhibiting the trunk cell potential of nude mice in the body of glue maternal cell tumors [43]. However, the therapeutic effect of SSD in iCCA and the underlying mechanisms have not been reported to date. Here, we explored SSD as a potential compound that may reverse gemcitabine resistance in iCCA. We found that a relatively lower dose of SSD (2.5 and 5 μM) can markedly enhance the cytotoxic effect of gemcitabine on RBE and HuCCT1 cells. Furthermore, this effect was found to be associated with the reversal of the ADRB2/glycolysis signaling pathway.

Nevertheless, there are some limitations in this study. We did not investigate whether ADRB2/glycolysis signaling can mediate gemcitabine resistance in iCCA cells under chronic stress *in vivo*, and whether SSD can reverse this resistance. In addition, the mechanism of SSD-mediated inhibition of the expression of ADRB2 at the mRNA level was not explored. Previous studies have shown that in liver cancer cells, SSD may inhibit the expression of COX-2 through the P-STAT3/HIF-1α pathway and reduce the expressions of P-STAT3 and HIF-1α, which inhibits the proliferation of HCC SMMC-7721 cells [44]. Another study proved that in HCC, SSD controls HCC cell proliferation by inhibiting the phosphorylated signal transducer and activator of transcription 3 CCAAT/enhancer binding protein beta (p-STAT3/C/EBPβ) signaling pathway and inhibits cyclooxygenase-2 (COX-2) expression. The expression levels of P-EIF2α/EIF2α, ATF4 and Chop in HCC cells were significantly reduced [45]. In response to the hepatic toxicity caused by acetaminophen, SSD can significantly suppress the phosphorylation of nuclear factor kappa B (NF-κB) and signal transducer and activator of transcription 3 (STAT3) and reverse APAP-induced inflammation in the target genes of NF-κB, such as pro-inflammatory cytokines IL6 and Ccl2, and those of STAT3, such as Socs3, Fga, Fgb and Fgg [46]. Although SSD has not found the adjustment mechanism of ADRB2, studies have shown that SSD regulates some transcription factors of tumor cells and changes the level of MRNA, which also lays the foundation for discovering the regulatory mechanism by which SSD regulates ADRB2. Therefore, we will carry out a series of studies to answer these questions in the future.

Taken together, our data indicate that the ADRB2/glycolysis signaling pathway can induce gemcitabine resistance in iCCA cell lines under chronic stress (stimulated with E and NE). Saikosaponin D, an ADRB2 inhibitor, can significantly reverse this therapeutic resistance. This study provides evidence and a basis for a better understanding of the effects of external environmental factors on iCCA progression and suggests that ADRB2 blockers can be used to clinically control iCCA progression and improve treatment efficacy.
